# Pre-attentive and Attentive Auditory Event-related Potentials in Children With Attention-Deficit Hyperactivity Disorder and Autism

**DOI:** 10.1177/15500594241255499

**Published:** 2024-05-16

**Authors:** Ulrich Schall, Ross Fulham, Max Günther, Jessica Bergmann, Renate Thienel, Julie Ortmann, Natalie G Wall, Paula Gómez Álvarez, Anne-Marie Youlden

**Affiliations:** 1 Centre for Brain and Mental Health Research, Mater Hospital, 5982 The University of Newcastle, New South Wales, Australia; 2 EDUCARE Specialists Services, Charlestown, New South Wales, Australia; 3 Hunter Medical Research Institute, New Lambton, New South Wales, Australia; 4 Institute of Psychology, 14310 Otto Friedrich University of Bamberg, Bamberg, Germany; 5 School of Medicine and Public Health, 5982 The University of Newcastle, Callaghan, New South Wales, Australia; 6 Department of Behavioural and Cognitive Sciences, 81872 University of Luxembourg, Esch-sur-Alzette, Luxembourg; 7 Faculty of Science & Engineering, 4571 Southern Cross University, Lismore, New South Wales, Australia

**Keywords:** autism, attention-deficit hyperactivity disorder, mismatch negativity, phonemes, P3b

## Abstract

Abnormalities in auditory processing are believed to play a major role in autism and attention-deficit hyperactivity disorder (ADHD). Both conditions often co-occur in children, causing difficulties in deciding the most promising intervention. Event-related potentials (ERPs) have been investigated and are showing promise to act as potential biomarkers for both conditions. This study investigated mismatch negativity (MMN) using a passive listening task and P3b in an active auditory go/no-go discrimination task. Recordings were available from 103 children (24 females): 35 with ADHD, 27 autistic, 15 autistic children with co-occurring ADHD, and 26 neurotypical (NT) children. The age range considered was between 4 and 17 years, but varied between groups. The results revealed increases in the MMN and P3b amplitudes with age. Older children with ADHD exhibited smaller P3b amplitudes, while younger autistic children showed reduced MMN amplitudes in response to phoneme changes compared to their NT counterparts. Notably, children diagnosed with autism and ADHD did not follow this pattern; instead, they exhibited more similarities to NT children. The reduced amplitudes of phonetically elicited MMN in children with autism and reduced P3b in children with ADHD suggest that the two respective ERPs can act as potential biomarkers for each condition. However, optimisation and standardisation of the testing protocol, as well as longitudinal studies are required in order to translate these findings into clinical practice.

## Introduction

Autism and attention-deficit hyperactivity disorder (ADHD) are both neurodevelopmental disorders. ADHD frequently co-occurs with autism.^1^ However, the pharmacotherapeutic approaches for these populations are different. Antipsychotics are more effective in regulating affect and challenging behaviours (including self-harming) in autistic children,^2^ whereas psychostimulants are the first-line pharmacotherapy for ADHD.^3^ As a consequence, the clinical decision-making for treatment (ie, psychostimulants vs antipsychotics) becomes particularly difficult when autistic children present with co-occurring ADHD. Event-related potentials (ERPs), which are derived from time-locked electroencephalographic (EEG) recordings in response to a stimulus or event, might act as potential biomarkers for guiding clinical decision-making to determine the most promising pharmacotherapy.^4^

Children with autism most commonly present with attenuated mismatch negativity (MMN). MMN represents the difference between an ERP in response to a rare deviant tone or sound minus the ERP in response to a more frequent standard tone or sound. The latency of the MMN is usually between 180 and 230 milliseconds (ms) depending on the type of stimulus with faster onset in response to pure tones differing in pitch compared to those differing in stimulus duration or other sound characteristics.^5^

The magnitude of the MMN amplitude is also influenced by sound characteristics and oddball probabilities,^6^ thus making it challenging to compare individual studies due to design differences. In addition, ERP characteristics change longitudinally with brain development.^7^ For instance, MMN amplitudes increase during brain maturation.^8^ Hence, age is an important design factor when investigating ERPs in children. Furthermore, smaller MMN amplitude has been previously associated with poor cognitive ability.^9^ A meta-analysis and systematic review of MMN in children with autism consistently found reduced MMN amplitude in young autistic children compared to neurotypical (NT) children when using non-speech auditory stimuli; however, these findings were inconsistent for more complex sounds.^10^

While MMN is an ERP associated with an automatic and ‘pre-attentive’ process, ADHD has been linked to reduced P3b amplitudes when performing active attention tasks using an oddball paradigm.^11^ P3b occurs at a latency of approximately 280-380 ms and its amplitude is determined by factors such as oddball probability, age, and performance accuracy.^12^ Preliminary evidence suggested that P3b amplitude increases in response to psychostimulant treatment in ADHD,^13^ although there is limited evidence supporting the effect of psychostimulants on MMN generation processes.^14^

The current study investigated MMN and P3b in four distinct groups of children: (1) those diagnosed with autism, (2) individuals with ADHD, (3) autistic children with co-occurring ADHD, and (4) NT children. Children with the ages from 4 to 17 years were included in our study. We hypothesised that autistic children would show reduced MMN amplitudes and children with ADHD would exhibit reduced P3b amplitudes compared to NT children, whereas autistic children with co-occurring ADHD would present with reductions in both MMN and P3b amplitudes. Therefore, our objective was to investigate these potential ERP biomarkers to complement the clinical decision-making process.

## Materials and Methods

### Participants

Diagnoses were made by senior clinicians (ie, clinical child psychologists and child and adolescent psychiatrists) according to the DSM-IV diagnostic criteria for ADHD (inattentive and combined sub-types) and autistic spectrum disorders (ASD; ie, autistic disorder, Asperger's syndrome, and pervasive developmental disorder). The diagnosing clinicians were not part of the research team. Neurodiverse participants were recruited through psychological and psychiatric referral, while NT children were recruited from the community via social media advertisements. Exclusion criteria for all children included neurological (eg, epilepsy) or endocrine conditions, traumatic brain injury with sustained unconsciousness, hearing impairment, or low intellectual ability (intelligence quotient (IQ) < 70). In addition, NT children with a history of mental health issues or medical conditions at the time of recording the EEG were excluded.

### Procedure

The procedure closely followed that of Weismüller et al^8^ and lasted between 1.5 and 2 h. Children were comfortably seated approximately 110 cm away from a 19 cm diagonal television screen. While sitting still and watching a silent movie, children were presented with various auditory stimuli during a passive listening recording condition. Short movement breaks were provided between recording blocks. In order to ensure their attention, children were promised a small toy if they correctly answered a few simple questions about the movie at the conclusion of the EEG recording.

Auditory stimuli were presented through calibrated headphones while the children were watching the muted movie. Two types of oddball stimuli were presented at a stimulus onset asynchrony (SOA) of 500 ms with an equal probability of *P* = .073 in a pseudo-random sequence, together with frequent (standard) 1 kHz-tones of 50 ms duration at 80 dB sound pressure level (SPL) intensity: (1) pitch-oddballs with 1.2 kHz stimulus frequency and (2) duration oddballs with 100 ms stimulus duration. Each oddball was followed by a minimum of two standard stimuli. In total, 700 stimuli were presented in each 6-min recording block.

Two types of phonemes (/da/ and /ga/) were also presented at 800 ms SOA in two subsequent EEG recording blocks of 6-min duration each. Stimuli were presented in a pseudo-random order of frequent /da/ and infrequent /ga/ phonemes (*P* = .08).^15^ The voice-onset times between the first consonant and the following vowel were 20 ms for /da/ and 60 ms for /ga/. In total, 450 stimuli were presented in each recording block.

**Figure 1.**
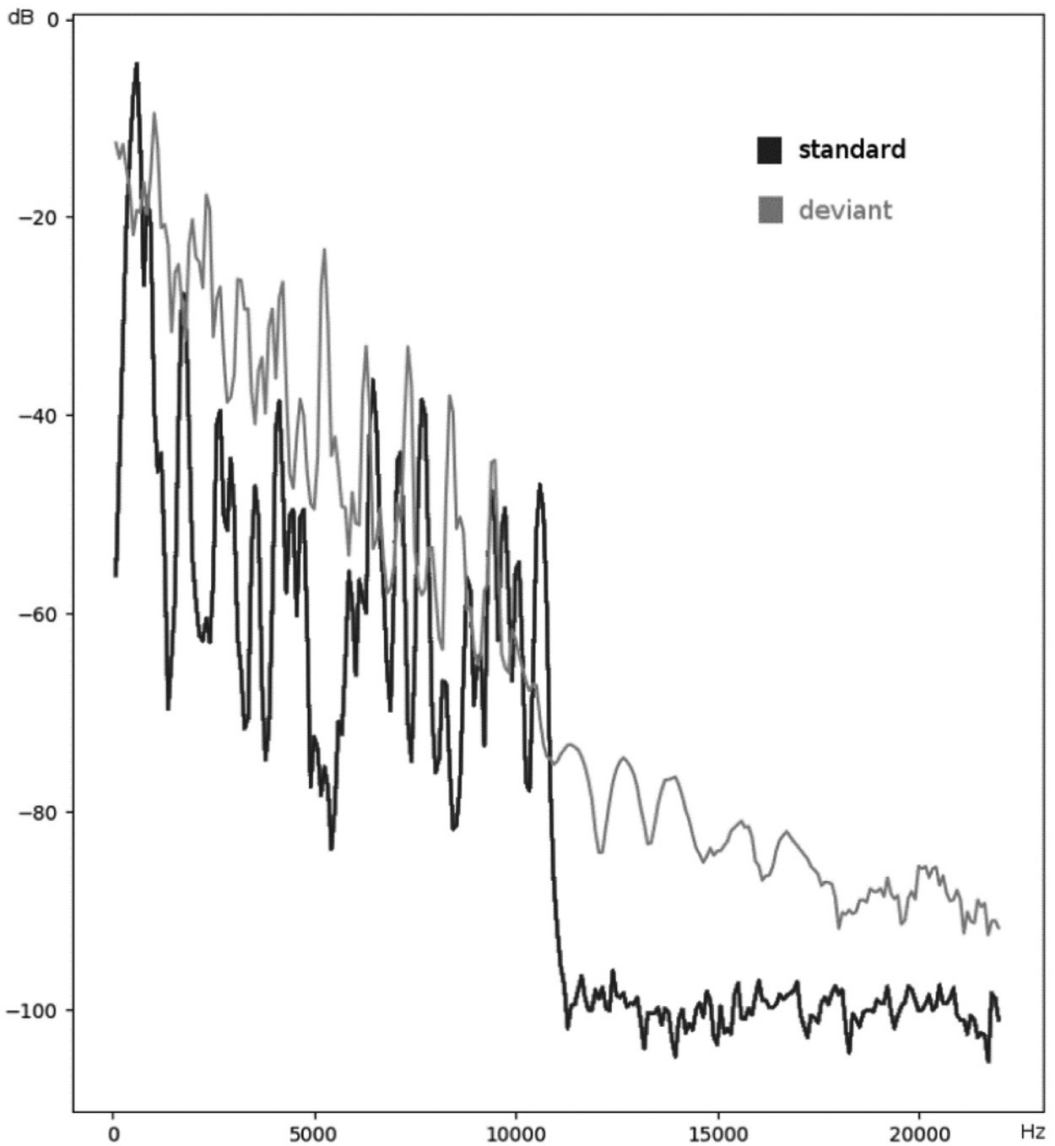
Frequency spectra of the standard and deviant sound stimuli in the P3b recording.

The recording of the P3b response mainly followed the procedure described by Price et al^16^ and took place after the completion of the passive listening recording blocks. Two 6-min recording blocks of 400 binaural stimuli each were presented through the headphones at an intensity of 80 dB SPL. The sound duration was 50 ms, with a rise and fall time of 10 ms. Standard and target stimuli were both complex sounds which did not differ in their average intensity, but had different frequency spectra (Figure 1). Standard sounds occurred with a probability of *P* = .9 and every target sound was followed by a minimum of two standard sounds. Stimuli were presented at a SOA of 1000 ms and the children were not watching a movie during this task. They were asked to press a button in response to the target sounds within 800 ms from the target sound onset. Children were given a short practice run prior to recording the EEG to ensure that they had understood the task. Button-press responses were also recorded during the EEG recording to monitor correct responses of >90%.

Continuous EEG data were recorded from the electrodes Fz, F3, F4, Cz, C3, C4, Pz, P3, and P4 (according to the International 10-20 System), as well as both mastoids against the nose reference (Neuroscan, El Paso, Texas, USA) using a 32-channels Quick-Cap (Compumedics Limited, Abbortsford, Victoria, Australia) equipped with Ag/AgCl-sintered electrodes. Vertical and horizontal eye movements were recorded above and below the left eye and 1 cm apart from each outer canthus. The sampling rate was 500 Hz and the EEG was filtered between 0.05 and 50 Hz for MNN recordings and between 0.1 and 30 Hz for P3b recordings. The impedance was kept below 5 kΩ at each electrode.

### EEG Analyses

The EEG were analysed offline using EEGDisplay v6.4.4.^17,18^ After visual inspection and removal of gross artefacts, the EEG was re-referenced to averaged mastoids, corrected for eye-blinks, and segmented into epochs. The epochs consisted of intervals between 100 ms pre-stimulus and 450 ms post-stimulus for the MMN recording, and between 100 ms pre-stimulus and 800 ms post-stimulus interval for the P3b recording. Baseline correction was performed over a pre-stimulus interval of 100 ms. Epochs containing gross artefacts (ie, exceeding ±100 μV) were eliminated. Ocular eye blink reduction followed by employing Neuroscan Edit software for EEG- vertical electro-oculography recording (VEOG) covariance analysis. The method creates a linear regression model for a point-for-point subtraction of the eye blink artefact from the EEG.

MMN was derived by subtracting ERPs in response to standard stimuli from those in response to oddball stimuli. Mean MMN amplitudes at Fz were calculated within the following post-stimulus intervals: (1) 130 to 200 ms for frequency oddballs, (2) 170 to 230 ms for duration oddballs, (3) 170 to 250 ms for phoneme oddballs, and (4) 310 to 360 ms for the P3b target response at Pz. ERP peak latencies were detected within each of these respective post-stimulus intervals. The minimum number of epochs for averaging were 80 for MMN and 40 for P3b.

### Statistical Analyses

Initially, an analysis of covariance (ANCOVA) was planned to be used to test the research hypotheses. However, our data did not meet the ANCOVA assumptions of normality within the experimental groups and homogeneity of variance. Therefore, a robust version of ANCOVA was used.^19^

Data were explored to check for outliers using boxplot diagrams. Despite the presence of outliers, they were not considered extreme or frequent enough to bias the test values. To ensure the independence of the predictor and the covariate, an analysis of variance (ANOVA) was conducted with the diagnosis group as factor and age as the dependent variable. Because assumptions for the general linear model were not met, a robust version of the WRS2 R package was conducted, which uses a generalisation of Welch's method.^20^

The research hypotheses were tested using a robust version of ANCOVA from the same R package. This approach compares trimmed means at up to five data points of the age covariate while making no parametric assumptions.^21^ Neither assumptions of homoscedasticity nor of normality or linearity of regression line have to be met. A running interval smoother was used to approximate regression lines. Up to five appropriate design points were chosen to form age cohorts for group comparison (ie, patients vs NT subjects).^19^ Design points were considered appropriate if the relationship between the dependent variable and the age covariate was similar enough in both groups, thereby controlling for age. Thus, instead of assuming a constant relationship between the age covariate and the dependent variable, this method searches for points where the slopes are approximately the same.^22^ Since an individual subject can be a member of more than one age cohort (ie, cohorts are indexed by their mean age), Bonferroni α-corrections were performed.

## Results

The sample with valid data consisted of 82 males and 21 females aged between 4 and 17 years old. Table 1 provides an overview of group size, age, and gender distribution. Some children were medicated at the time of the EEG recording. Specifically, two autistic children were treated with atomoxetine and clonidine. Among the children with ADHD, 14 were treated with methylphenidate, four with atomoxetine, and two with clonidine. Two autistic children with co-occurring ADHD were treated with risperidone, one with atomoxetine, and two with clonidine.

**Table 1.** Group Characteristics of Autistic (ASD), ADHD, Neurotypical Children (NT) and Autistic Children with co-Morbid ADHD (ASD + ADHD). Wilcox Robust Version of an ANOVA was Used and Chi-Square for Gender (Female = f).

	Groups				ANOVA		
	NT	ASD	ADHD	ASD + ADHD	F	df (1;2)	*P*
Age (SD)	10.09 (2.48)	8.89 (3.37)	9.17 (2.76)	9.13 (2.97)	1.695	3; 27.64	.191
Gender	n = 26 (11f)	n = 27 (6f)	n = 35 (6f)	n = 15 (1f)	Chi2=	8.33	.039

ADHD, attention-deficit hyperactivity disorder; NT, neurotypical; ASD, autistic spectrum disorder; ANOVA, analysis of variance.

**Figure 2.**
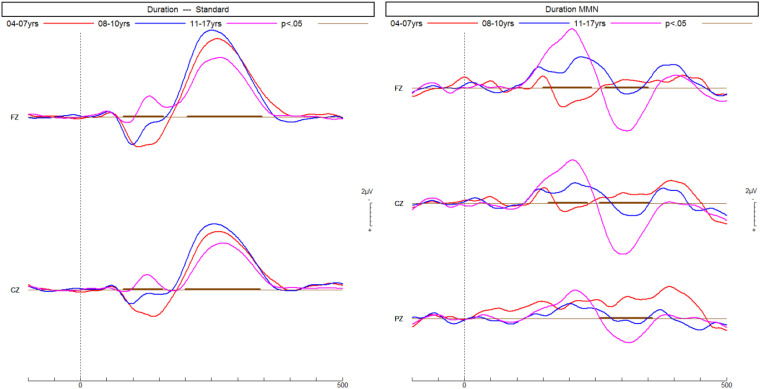
ERPs of neurotypical children recorded in a passive listening task increase with age in response to the frequent standard tone and the infrequent longer duration deviant tone, thus resulting in a larger MMN predominantly at the front-central electrode Fz. ERPs, event-related potentials. MMN, mismatch negativity.

**Figure 3.**
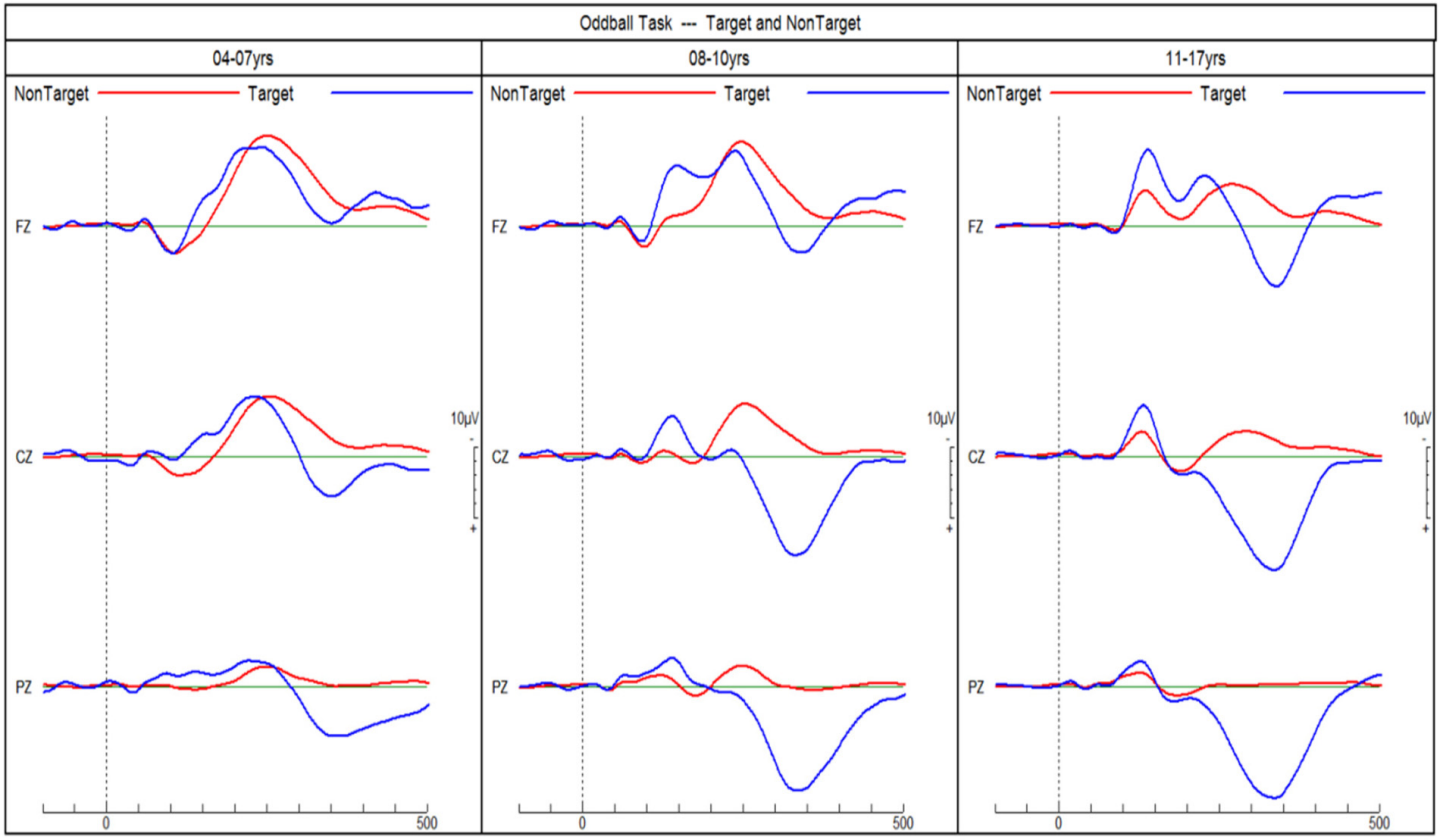
ERPs of neurotypical children in response to target and non-target tones in an auditory go/no-go discrimination task. P3b amplitudes in response to target tones increase with age. ERPs, event-related potentials.

Age did not differ between the diagnostic groups (Table 1), but was correlated (Spearman) with larger mean ERP amplitudes across all groups: frequency MMN r = −0.32 (*P* < .01); duration MMN r = −0.55 (*P* < .001); phonetic MMN r = −0.35 (*P* < .01); P3b r = 0.38 (*P* < .001). Figures 2 and 3 show the increase of MMN and P3b amplitudes with age in NT children, respectively. Only phonetic MMN was significantly correlated with age in the NT group (r = −0.66, *P* < .01). Hence, the group means were compared for individual age cohorts with sufficient data.

**Figure 4.**
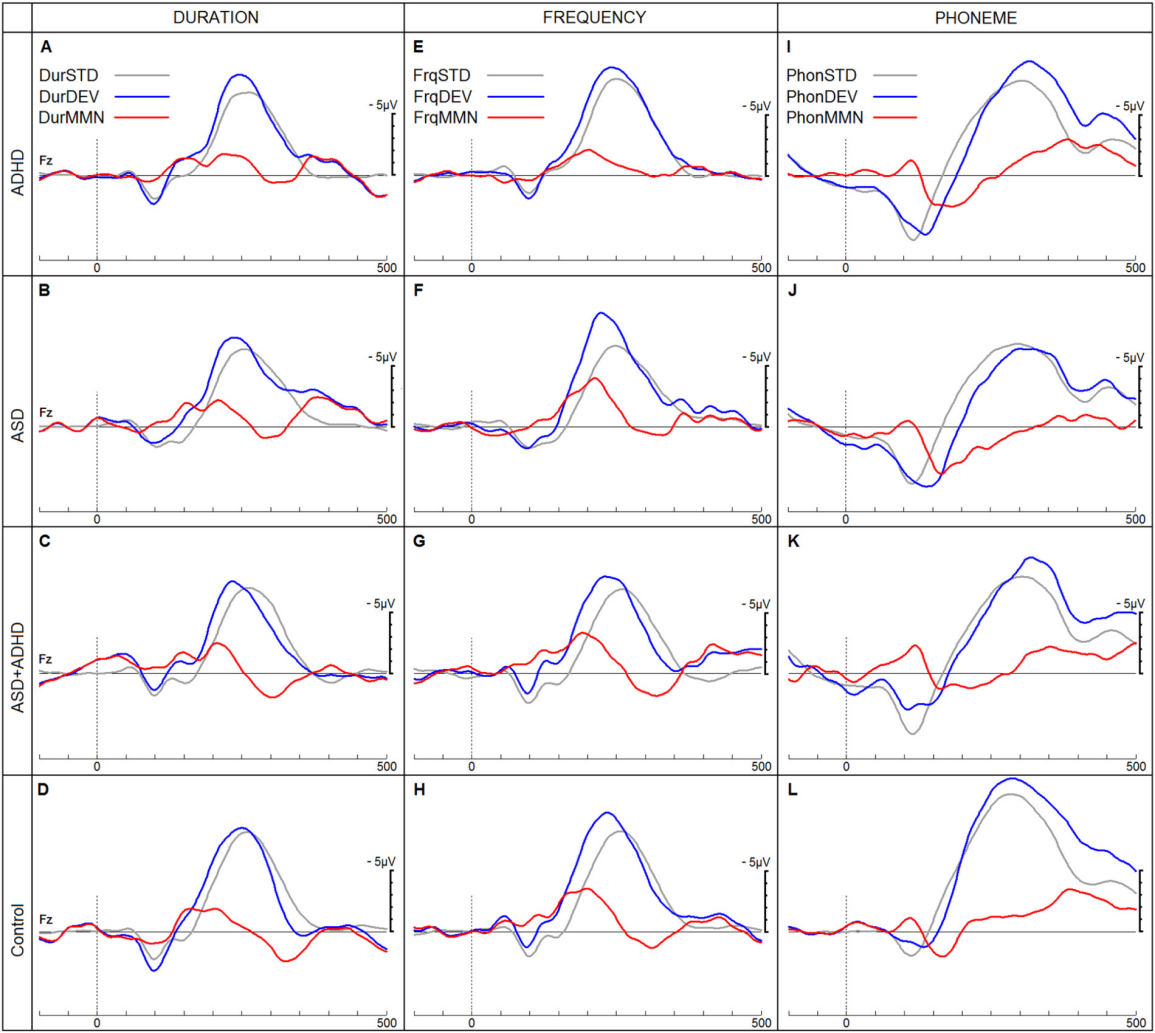
ERPs in response to duration (Dur), frequency (Frq) and phoneme (Phon) stimuli in a passive listening task. ERPs in response to standard (STD), deviant (DEV) and resulting MMN are shown for the four groups (Attention-deficit Hyperactivity Disorder (ADHD), Autistic Spectrum Disorder (ASD), Autistic Spectrum Disorder with co-morbid ADHD and neurotypical children). ERPs, event-related potentials; MMN, mismatch negativity.

**Figure 5.**
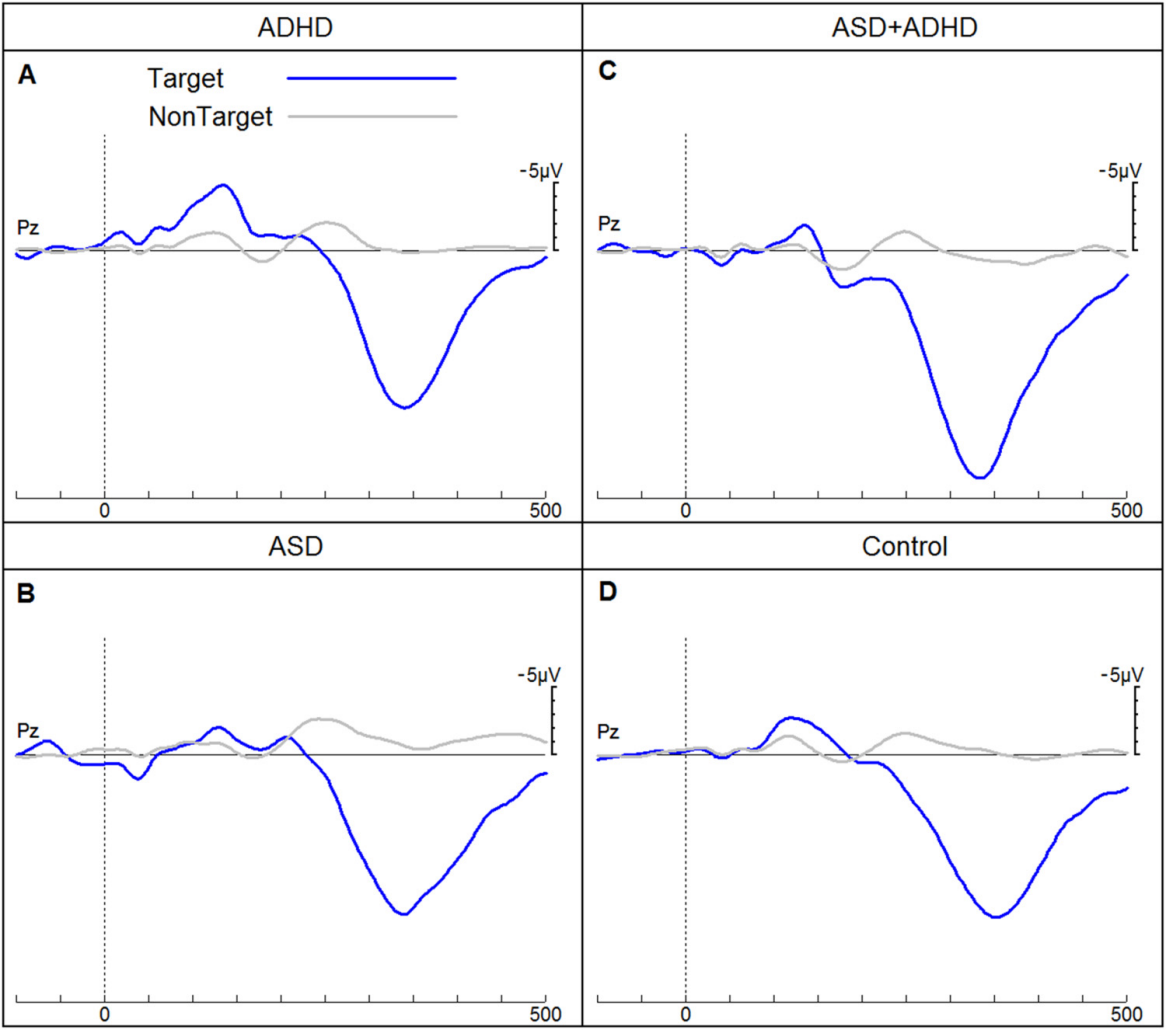
ERPs of children for the four groups (attention-deficit hyperactivity disorder (ADHD), autistic spectrum disorder (ASD), autistic spectrum disorder with co-morbid ADHD and neurotypical children) in response to target and non-target tones elicited in an auditory go/no-go discrimination task. ERPs, event-related potentials.

Figure 4 shows the three different MMN ERPs and Figure 5 shows the P3b ERPs for each of the four groups. The subsequent sections report the findings for each diagnostic group and age cohort.

### Autistic Children

Only frequency MMN (r = −0.44, *P* < .5) and duration MMN (r = −0.56, *P* < .01) were significantly correlated with age in the autistic group. In the phonetic condition, the amplitude of the MMN response at Fz (170-250 ms) was significantly smaller in children with autism than in NT children for the 7.9- and 10.4-year age cohorts (Table 2). No significant differences were found between NT and autistic children for either mean amplitude at Fz (130-200 ms) or peak latency of the MMN in response to frequency deviants. Autistic children in the 7.9-year age cohort showed a significantly shorter latency in their MMN response to duration deviants. Group differences in latency in the 9-year-old group and mean amplitude in all age cohorts were not significant (Table 3). Regarding the P3b response to target stimuli, autistic children did not differ significantly from their NT counterparts (Table 4).

**Table 2.** Results of Wilcox’ Robust ANCOVA for Differences in Phoneme MMN Mean Amplitude (170-250 ms) at Fz Between Neurotypical (NT) and Autistic Children (ASD) by Age Cohorts.

Mean Group Age	n NT	n ASD	Difference	SE	CI	F	*P*
7.9	13	17	−3.536	1.029	−6.483; −0.590	3.435	.003*
9.7	17	18	−2.545	1.039	−5.466; 0.376	2.449	.023
10.4	17	18	−2.660	0.948	−5.320; 0.001	2.805	.011*
11.6	17	12	−2.531	0.977	−5.347; 0.285	2.590	.020

Annotation: Differences in µV; SE = standard error; CI = confidence interval; * = significant difference with α < .0125 (Bonferroni-corrected).

MMN, mismatch negativity; NT, neurotypical; ASD, autistic spectrum disorder; ANCOVA, analysis of covariance.

**Table 3.** Results of Wilcox’ Robust ANCOVA for Differences in Duration MMN Mean Amplitude (170-230 ms) and Peak Latency at Fz Between Neurotypical (NT) and Autistic Children (ASD) by Age Cohorts.

Mean Group Age	n NT	n ASD	Parameter	Difference	SE	CI	F	*P*
7.9	15	13	Amplitude	1.091	1.491	−3.246; 5.429	0.732	.475
			Latency	29.152	10.554	−1.664; 59.969	2.762	.015*
8.5	14	17	Amplitude	−0.283	1.392	−4.227; 3.662	0.203	.841
			Latency	20.691	9.571	−6.669; 48.051	2.162	.048
9.6	13	17	Amplitude	−1.778	1.579	−6.364; 2.806	1.126	.277
			Latency	10.529	10.413	−19.200; 40.257	1.011	.326

Annotation: Differences in µV for amplitude and ms for latency; SE = standard error; CI = confidence interval; * = significant difference with α < .017 (Bonferroni-corrected).

MMN, mismatch negativity; NT, neurotypical; ASD, autistic spectrum disorder.

**Table 4.** Results of Wilcox’ Robust ANCOVA for Differences in P3b Mean Amplitude (310-360 ms) and Peak Latency to Target Stimuli at Pz Between Neurotypical (NT) and Autistic Children (ASD) by Age Cohorts.

Mean Group Age	n NT	n ASD	Parameter	Difference	SE	CI	F	*P*
8.2	17	8	Amplitude	−0.886	3.718	−11.970; 10.199	0.238	.816
			Latency	−3.470	15.672	−50.387; 43.458	0.221	.828
9.6	19	11	Amplitude	−1.473	2.998	−10.022; 7.075	0.491	.629
			Latency	0.149	17.913	−52.103; 52.401	0.008	.994
10.7	18	12	Amplitude	0.570	2.540	−6.703; 7.842	0.224	.825
			Latency	−7.360	14.984	−50.273; 35.552	0.491	.630
11.4	12	8	Amplitude	2.942	2.721	−5.283; 11.267	1.081	.303
			Latency	−17.842	16.274	−66.844; 31.160	1.096	.295
13.1	10	6	Amplitude	4.335	1.722	−1.384; 10.054	2.518	.038
			Latency	1.447	11.079	−43.793; 46.687	0.131	.902

Annotation: Differences in µV for amplitude and ms for latency; SE = standard error; CI = confidence interval; significant difference with α < .01 (Bonferroni-corrected).

NT, neurotypical; ASD, autistic spectrum disorder; ANCOVA, analysis of covariance.

### Children with ADHD

The ADHD group showed a similar age correlation pattern as the autistic group (frequency MMN: r = −0.44, *P* < .05; duration MMN: r = −0.61, *P* < .001). The analysis did not reveal a significantly smaller mean amplitude (170-250 ms) at Fz of the MMN response to phoneme oddballs in children with ADHD compared to NT children (Table 5). The MMN mean amplitude (130-200 ms) and peak latency in response to frequency deviants did not significantly differ between the two groups. Peak latencies of MMN in response to duration deviants at Fz did not differ significantly between NT and ADHD children. No MMN mean amplitude differences (170-230 ms) were observed between groups (Table 6).

**Table 5.** Results of Wilcox’ Robust ANCOVA for Differences in Phoneme MMN Mean Amplitude (170-250 ms) at Fz Between Neurotypical Children (NT) and Children With ADHD by Age Cohorts.

Mean Group Age	n ADHD	n NT	Difference	SE	CI	F	*P*
7.9	22	13	2.044	0.947	−0.613; 4.701	2.160	.043
8.6	22	17	1.818	1.008	−0.986; 4.622	1.804	.084
9.8	24	17	1.160	0.915	−1.380; 3.700	1.268	.217
11.2	13	17	1.345	1.056	−1.690; 4.380	1.274	.220

Annotation: Differences in µV; SE = standard error; CI = confidence interval; significant difference with α < .0125 (Bonferroni-corrected).

ADHD, attention-deficit hyperactivity disorder; MMN, mismatch negativity; NT, neurotypical; ANCOVA, analysis of covariance.

**Table 6.** Results of Wilcox’ Robust ANCOVA for Differences in Duration MMN Mean Amplitude (170-230 ms) and Peak Latency at Fz Between Neurotypical Children (NT) and Children With ADHD by Age Cohorts.

Mean Group Age	n ADHD	n NT	Parameter	Difference	SE	CI	F	*P*
7.9	23	13	Amplitude	1.141	1.374	−2.901; 5.183	0.830	.420
			Latency	18.533	7.819	−3.996; 37.280	2.169	.030
8.6	23	17	Amplitude	1.450	1.251	−2.097; 4.996	1.159	.261
			Latency	16.226	7.481	−4.828; 37.280	2.169	.042
9.0	25	17	Amplitude	0.616	1.193	−2.737; 3.970	0.517	.611
			Latency	9.900	8.636	−14.791; 34.591	1.146	.267
11.2	13	17	Amplitude	−1.745	1.525	−6.167; 2.678	1.144	.270
			Latency	5.055	7.900	−17.502; 27.611	0.640	.531

Annotation: Differences in µV for amplitude and ms for latency; SE = standard error; CI = confidence interval; significant difference with α < .0125 (Bonferroni-corrected).

ADHD, attention-deficit hyperactivity disorder; MMN, mismatch negativity; NT, neurotypical; ANCOVA, analysis of covariance.

Children with ADHD exhibited a significantly smaller mean amplitude (310-360 ms) in their P3b response to target stimuli at Pz than NT children in the 11.4-year age cohort. There was a tendency for longer peak latencies at the age cohort of 8 years. The mean amplitude and peak latency did not differ between the ADHD and NT groups in all age cohorts (Table 7).

**Table 7.** Results of Wilcox’ Robust ANCOVA for Differences in P3b Mean Amplitude (310-360 ms) and Peak Latency to Target Stimuli at Pz Between Neurotypical Children (NT) and Children With ADHD by Age Cohorts.

Mean Group Age	n ADHD	n NT	Parameter	Difference	SE	CI	F	*P*
8.1	21	8	Amplitude	0.684	3.381	−9.672; 11.040	0.202	.844
			Latency	−29.053	12.325	−72.041; 13.934	2.357	.055
9.3	24	11	Amplitude	−0.210	2.611	−7.659; 7.238	0.081	.937
			Latency	−14.156	13.770	−60.880; 32.570	1.028	.339
10.2	20	12	Amplitude	1.487	2.585	−5.904; 8.879	0.575	.572
			Latency	−12.475	11.155	−47.978; 23.029	1.118	.293
11.4	12	8	Amplitude	4.855	3.035	−4.294; 14.003	1.600	.136
			Latency	−9.645	11.112	−47.368; 28.079	0.868	.415
13.2	10	6	Amplitude	7.997	2.190	0.465; 15.529	3.652	.009*
			Latency	10.557	12.879	−34.082; 55.195	0.820	.442

Annotation: Differences in µV for amplitude and ms for latency; SE = standard error; CI = confidence interval; * = significant difference with α < .01 (Bonferroni-corrected).

ADHD, attention-deficit hyperactivity disorder; NT, neurotypical; ANCOVA, analysis of covariance.

In order to test the potential medication effects on P3b mean amplitude, a comparison was conducted between 14 methylphenidate-medicated children with ADHD and 14 age-matched unmedicated children with ADHD. No significant differences were observed between age groups (Mann-Whitney U-Test: *P* = .4) or P3b mean amplitude (Mann-Whitney U-Test: *P* = .32).

### Autistic Children with Co-Occurring ADHD

Age was significantly correlated in children diagnosed with ASD and ADHD: phonetic MMN (r = −0.49, *P* < .05), duration MMN (r = −0.57, *P* < .05), and P3b mean amplitudes (r = 0.54, *P* < .05). The ANCOVA analysis revealed no group differences between autistic children with co-occurring ADHD and NT children in their MMN mean amplitude and peak latencies at Fz for phonetic, frequency, and duration stimuli in all age cohorts. The P3b response to targets at Pz in autistic children with ADHD exhibited a trend of significantly shorter latencies than those measured in NT children in the 8.1- and 10.5-year age cohorts (Table 8). There were no significant differences in mean amplitude (310-360 ms).

**Table 8.** Results of Wilcox’ Robust ANCOVA for Differences in P3b Mean Amplitude (310-360 ms) and Peak Latency to Target Stimuli at Pz Between Neurotypical Children (NT) and Autistic Children With ADHD (ASD/ADHD) by Age Cohorts.

Mean Group Age	n NT	n ASD/ADHD	Parameter	Difference	SE	CI	F	*P*
8.1	8	10	Amplitude	−3.430	4.338	−16.489; 9.629	0.791	.448
			Latency	43.900	15.508	−2.460; 90.260	2.831	.018
9.3	11	9	Amplitude	−2.773	3.632	−13.709; 8.163	0.763	.464
			Latency	32.949	17.407	−17.567; 83.464	1.893	.083
10.5	12	7	Amplitude	−2.556	3.304	−13.167; 8.055	0.773	.464
			Latency	34.107	12.550	−2.772; 70.985	2.718	.020
11.2	8	7	Amplitude	−3.675	3.473	−14.629; 7.278	1.058	.322
			Latency	25.846	12.297	−12.209; 63.901	2.102	.067

Annotation: Differences in µV for amplitude and ms for latency; SE = standard error; CI = confidence interval; significant difference with α < .0125 (Bonferroni-corrected).

ADHD, attention-deficit hyperactivity disorder; NT, neurotypical; ASD, autistic spectrum disorder; ANCOVA, analysis of covariance.

## Discussion

This study examined MMN and P3b ERPs in children with autism, ADHD, and autism with comorbid ADHD. In the NT group, only MMN amplitude in response to phoneme deviants significantly increased with age, indicating improved phoneme deviant detection with greater brain maturity. However, no significant age-dependent mean amplitude changes were observed for duration MMN, frequency MMN, or P3b. This lack of significance suggests that the underlying auditory change and target detection processes may not experience substantial developmental changes during brain maturation (beyond phonetic MMN) in the age range investigated here.^23^ Nevertheless, a longitudinal study that incorporates other measures of brain maturation and cognitive ability is required to support this interpretation.

Children with autism presented with smaller MMN amplitudes in response to phoneme deviants compared to NT children. Thus, this amplitude reduction did not correlate with age, suggesting an autism-specific lack of neurodevelopmental changes in phoneme deviant detection. However, frequency and duration of MMN amplitudes increased with age, indicating additional gains in less complex change detection with brain neurodevelopment.

The existing literature on MMN amplitudes is inconsistent. For instance, previous studies have shown similar reductions in MMN amplitudes in response to phoneme deviants,^24,25^ contrary to the results reported by Yu et al^26^ Other studies have also reported smaller MMN in response to duration^27,28^ and frequency deviants.^29,30^ Nevertheless, consistent with the findings of this study, Gomot et al^31^ we did not find MMN amplitude reductions in autistic children with frequency deviants. Some studies have even found larger phonetically elicited MMN amplitudes in children with autism.^32,33^ These inconsistencies are partly explained by differences in methodology, variations in sound characteristics, small sample sizes, and heterogeneity within the groups studied, such as autistic children with other co-occurring diagnoses. In addition, the diagnostic criteria for autism have changed over time.

Age-dependent amplitude increases were observed in the ADHD group for frequency and duration MMN, while phonetic MMN and P3b amplitudes did not considerably differ across the age ranges investigated here. P3b mean amplitudes were smaller in children with ADHD when compared to NT children with a mean age of 11.4 years. These findings suggest that auditory change detection improves in children with ADHD as their brains mature, but they increasingly fall behind their NT counterparts in their auditory target detection when getting older. However, psychostimulant pharmacotherapy may have masked the P3b amplitude reduction in younger children with ADHD.^34^ This possibility was not confirmed in the present study.

The smaller P3b amplitudes observed in children with ADHD have been consistently reported in other studies.^35–39^ In addition, Rothenberger et al^40^ reported an MMN reduction in response to pure tone deviants, while other studies did not replicate this finding.^41–43^

The pattern of ERP findings in autistic children with co-occurring ADHD differed from those in the other clinical groups. While target detection (P3b), duration, and phonemic change detection (MMN) appeared to improve with increased brain maturity, the mean amplitude measures of all ERPs did not significantly differ from those of NT children across all age groups. This indicates that the comorbid group exhibited distinct ERP patterns, rather than a combination of the ERP changes observed in the autistic and ADHD groups, as hypothesised.

## Limitations

Further investigation of the clinical presentations and associations between ERPs and symptoms, instead of just relying on diagnoses, would provide valuable insights. However, such a level of detail was not consistently available in our study, as it relied on existing data.

Moreover, our study used a clinical convenience sample that lacked a systematic recruitment process, thus limiting the ability to extrapolate these findings to the rest of the clinical population. In addition, the variability in age composition within groups has limited the statistical analyses due to the reduced sample size in the age subgroups. Also, the lack of access to the patients’ clinical files, which was a requirement imposed by our ethics committee to protect the children's anonymity, limited a more detailed exploration of the data.

## Conclusion and Future Directions

Despite these limitations, the consistent observations of diminished phonetically elicited MMN amplitudes highlight its potential as a biomarker in autistic children. Further optimisation and standardisation of the testing protocol are required in order to translate these findings into clinical practice, where they could offer valuable diagnostic assistance and intervention guidance.

Similarly, the consistent observations of P3b amplitude reductions in children with ADHD signify its potential as biomarker for this condition. However, it is currently premature to consider MMN and P3b for clinical applications. The recording methods require more refinement to be sufficiently reliable for a clinical use. Continued research efforts aimed to refine these ERP methodologies are needed to further explore their clinical utility.
